# Thoracic Synovial Cyst at the Th2-3 Level Causing Myelopathy

**DOI:** 10.1155/2017/6257294

**Published:** 2017-09-07

**Authors:** Martin M. Sundskarð, Shahin Gaini

**Affiliations:** ^1^Medical Department, National Hospital Faroe Islands, Tórshavn, Faroe Islands; ^2^Infectious Diseases Research Unit, Odense University Hospital and University of Southern Denmark, Odense, Denmark; ^3^Department of Science and Technology, University of the Faroe Islands, Tórshavn, Faroe Islands

## Abstract

Intraspinal synovial cyst is a rare cause of myelopathy. These cysts present most often in the lumbar and cervical parts of the spine but are more infrequent in the thoracic spine. We present a case of a 73-year-old man with an intraspinal, extradural synovial cyst at the Th2-3 level causing paraesthesia and weakness in the legs. A laminectomy and excision of the cyst were performed and the patient recovered fully. In the thoracic spine, synovial cysts are almost exclusively found in the lower part. Laminectomy, with excision, is the treatment of choice, although steroid injections have been described.

## 1. Introduction

Intraspinal synovial and ganglion cysts are a rare cause of spinal cord compression [1]. They often arise from degenerative joints in the spinal column but the etiology is yet unknown [1–4]. The cysts are most often found in the more mobile parts of the spine, in the lumbar and cervical segments. They are rarely found in the thoracic spine [5]. We present a case of an intraspinal cyst compressing the medulla at the Th2-3 level.

## 2. Case

A 73-year-old man, with a past history of autoimmune hepatitis, presented to the hospital with progressing neurological symptoms. He had, in over a period of two months, developed ascending paraesthesia, abnormal gait, weakness in the legs, and trouble controlling vesical and anal sphincters. Physical exam revealed paraesthesia at the level of the nipple (Th-4 level) and in the legs. In the lower extremity there was bilateral weakness at the hip and knee joint as well as dorsal flexion of the foot.

**Figure 1:**
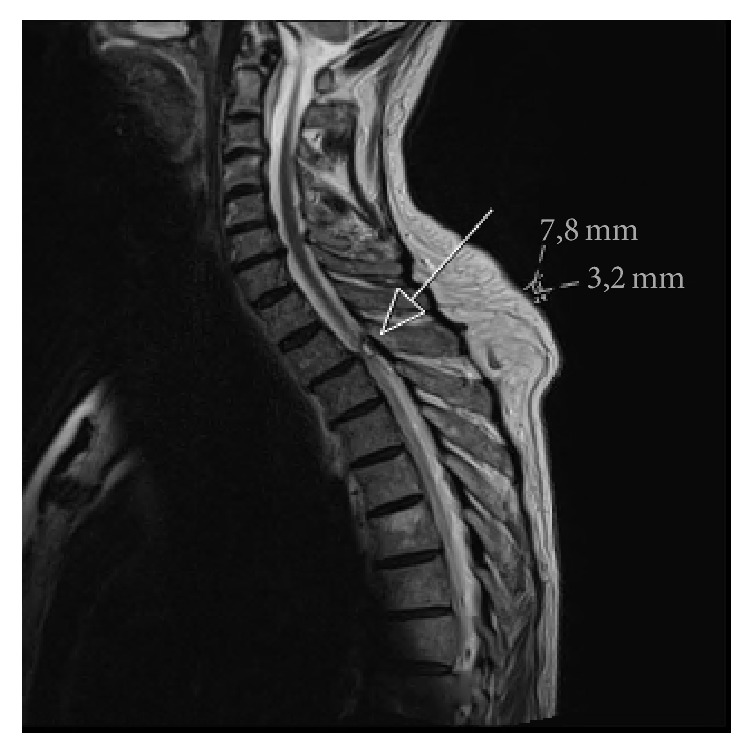
MRI of the upper spinal column T2-weighted sequence, sagittal plane. White arrow showing a 7,8 mm by 3,2 mm cyst at the Th2-3 level on the right side.

**Figure 2:**
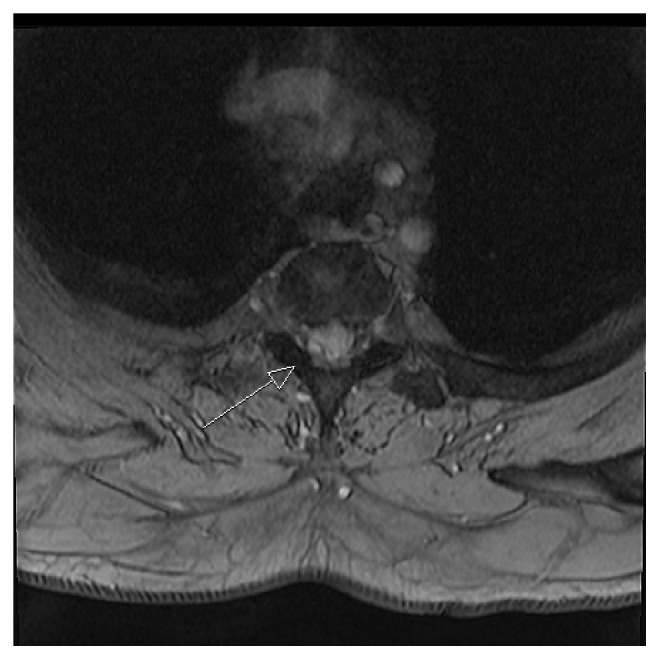
MRI of the upper spinal column T2-weighted sequence, transverse plane. White arrow indicating tumor location with compression of spinal cord.

Suspecting spinal stenosis an MRI of the spine was ordered. This showed a 4 × 8 mm intraspinal, extradural cystic process at the Th2-3 level on the right side (Figures 1 and 2). The cyst was white on T2 weighted imaging and showed no change in signal with infusion of contrast. The radiologist assessed the cyst to be congruent with a ganglion.

The patient was started on oral prednisolone 75 mg per day and transferred to Iceland for surgery. A decompressive laminectomy was performed and the cyst was excised. The operating surgeon found the operative material congruent with a synovial cyst; it was however not sent for pathological study. The patient had immediate improvement of the symptoms. Within a few days he was able to walk unassisted. Over a period of six weeks he regained his full strength; only the paraesthesia remained.

The patient was seen 2.5 years after surgery where he reported that the paraesthesia had remitted completely but it had taken a couple of years.

## 3. Discussion

We present a case of an elderly man, with no history of degenerative disease of the spine or trauma, with a cyst in the upper part of the thoracic column. It is unclear how long the patient had the cyst; probably it could have been there for years. He presented with symptoms progressing over a period of a few weeks, primarily paraesthesia with some weakness of the lower limbs.

Intraspinal cysts occur mostly in the lumbar segments; they are very rarely seen in the thoracic part of the spine [5] and account for only about 1% of cysts involving the columna vertebralis. In one large study, thoracic cysts accounted for only 0,06% (9 cases out of 16,000 cases) of all decompressive spinal surgeries over a 27-year period. None of these 9 cysts were located above the Th10-level [1].

To our best knowledge only 25 cases describing synovial or ganglion cysts in the thoracic region have been reported [1, 2, 4–8].

The prevalence of intraspinal cysts is to our knowledge unknown. There are many case reports and case series, but no long-term observational studies. It is unlikely we will ever know the true prevalence, because of the rarity of the condition and the fact that an MRI is required to diagnose it.

The treatment for synovial cysts consists mostly of a decompressive laminectomy and excision of the cyst. In a few cases conservative treatment has been tried. In one series four patients had significant improvement with steroid injections, although the relief was only short term. In one case the cyst regressed completely with injection; however pain persisted due to degenerative disease [3]. With surgical treatment approximately 90% of patients experience almost complete resolution of symptoms [1, 4, 5]. Those who have less improvement usually also have more degenerative disease and a long history of back pain [3, 4].

## 4. Conclusion

Intraspinal synovial cysts of the thoracic spine are a very rare cause of medullary compression. They are mostly found in the lower part of the thoracic column. MRI of the spine is the gold-standard imaging modality for both diagnostics and planning the surgical procedure. Most often the treatment consists of laminectomy and excision, but conservative treatment with steroid injection has been described. Clinicians should be aware of this rare cause of paraesthesia and weakness of the lower limbs. Acute neurosurgical intervention can reduce the risk of irreversible neurological damage of the medulla spinalis.
